# Crocin Protects the 661W Murine Photoreceptor Cell Line against the Toxic Effects of All-*Trans*-Retinal

**DOI:** 10.3390/ijms251810124

**Published:** 2024-09-20

**Authors:** Bo Yang, Kunhuan Yang, Jingmeng Chen, Yalin Wu

**Affiliations:** 1 Fujian Provincial Key Laboratory of Ophthalmology and Visual Science, Fujian Engineering and Research Center of Eye Regenerative Medicine, Eye Institute of Xiamen University, School of Medicine, Xiamen University, Xiamen 361102, China; 2 School of Medicine, Xiamen University, Xiamen 361102, China; 3 Shenzhen Research Institute of Xiamen University, Shenzhen 518057, China

**Keywords:** crocin, photoreceptor, all-*trans*-retinal, apoptosis, pyroptosis, ferroptosis

## Abstract

Age-related macular degeneration (AMD) is a common disease contributing to vision loss in the elderly. All-*trans*-retinal (atRAL) is a retinoid in the retina, and its abnormal accumulation exhibits toxicity to the retina and promotes oxidative stress-induced photoreceptor degeneration, which plays a crucial role in AMD progression. Crocin is a natural product extracted from saffron, which displays significant antioxidant and anti-inflammatory effects. The present study elucidates the protective effects of crocin on photoreceptor cell damage by atRAL and its potential mechanisms. The results revealed that crocin significantly attenuated cytotoxicity by repressing oxidative stress, mitochondrial injury, and DNA damage in atRAL-loaded photoreceptor cells. Moreover, crocin visibly inhibited DNA damage-induced apoptosis and gasdermin E (GSDME)-mediated pyroptosis in photoreceptor cells after exposure to atRAL. It was also observed that crocin distinctly prevented an increase in Fe^2+^ levels and lipid peroxidation caused by atRAL via suppressing the Kelch-like ECH-associated protein 1 (KEAP1)/nuclear factor-erythroid 2-related factor 2 (NRF2)/heme oxygenase-1 (HO-1) signaling pathway, thereby ameliorating photoreceptor cell ferroptosis. In short, these findings provide new insights that crocin mitigates atRAL-induced toxicity to photoreceptor cells by inhibiting oxidative stress, apoptosis, pyroptosis, and ferroptosis.

## 1. Introduction

Age-related macular degeneration (AMD) is a prevalent condition among the elderly, characterized by photoreceptor damage on the macular region of the retina, leading to a gradual decline in central vision [1]. AMD is divided into two categories: wet AMD, which is characterized by retinal neovascularization, and dry AMD, which is characterized by retinal geographic atrophy [2]. The treatment for wet AMD is mainly anti-angiogenic therapy, while there is no effective treatment for dry AMD [3]. Currently, approximately 200 million individuals are diagnosed with AMD in the world, with projections indicating a rise to nearly 300 million by 2040 [4]. This trend underscores the growing significance of AMD as a major public health issue.

All-*trans*-retinal (atRAL), a retinoid in the visual cycle, must be cleared in a timely manner to maintain normal visual function [5]. The abnormal accumulation of atRAL in photoreceptors due to the disrupted visual cycle is closely associated with the pathogenesis of dry AMD [6]. There is already evidence that atRAL promotes apoptosis via oxidative stress, DNA damage, mitochondrial injury, and endoplasmic reticulum stress and drives pyroptosis via the activation of gasdermin E (GSDME) in a murine cone photoreceptor cell line 661W [7,8,9]. Also, it has been found that atRAL provokes iron dyshomeostasis and glutathione (GSH) depletion and elicits ferroptosis in 661W cells through the activation of heme oxygenase-1 (HO-1) [10,11]. The decreased activity of antioxidant systems is considered to be a non-genetic risk factor for dry AMD [2,3,4,5,6,7,8,9,10,11,12].Given that oxidative stress caused by atRAL plays an important role in photoreceptor cell death [7], the timely supplementation of antioxidants may alleviate photoreceptor cell damage and delay the progression of dry AMD.

Crocin, a water-soluble carotenoid derived from saffron crocus flowers, is a natural product traditionally used as a coloring and flavoring agent [13,14]. It exhibits a range of pharmacological effects, notably antioxidant and anti-inflammatory properties [14,15]. Crocin has demonstrated potential efficacy in animal models of glaucoma and retinal ischemia/reperfusion injury [16,17]. Moreover, a clinical trial indicated that the short-term administration of crocin alleviates retinal damage in diabetic maculopathy [18]. However, the effects of crocin on dry AMD remain to be clarified.

In the present study, we elucidated the role of crocin in attenuating photoreceptor damage by atRAL and its underlying mechanisms with a focus on inhibitory effects on oxidative stress, apoptosis, pyroptosis, and ferroptosis.

## 2. Results

### 2.1. Crocin Enhances Cell Viability and Alleviates Oxidative Stress in atRAL-Loaded 661W Cells

**Figure 1:**
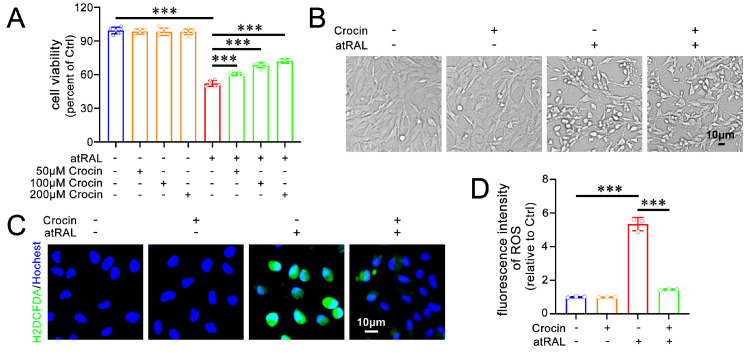
Effects of crocin on cell viability and oxidative stress of atRAL−loaded 661W cells. (**A**) Cell viability was assessed by MTS assay. (**B**) Microscopic photographs. (**C**) ROS production was visualized by H2DCFDA staining. (**D**) Fluorescence intensity reflecting ROS levels was quantified by ImageJ software (version 2.9.0), and shown as fold changes relative to vehicle (DMSO)-treated controls. Scale bars in (**B**,**C**), 10 μm. *** *p* < 0.001.

A previous study from our laboratory established an experimental model for studying the toxicity of atRAL in 661W cells [7]. The MTS assay was used to assess the protective effects of crocin in atRAL-loaded 661W cells. Cell viability significantly decreased when 661W cells were treated with 5 μM of atRAL alone for 6 h. Notably, treatment with crocin at different concentrations (50, 100, and 200 μM) significantly and concentration-dependently enhanced the viability of atRAL-treated 661W cells (Figure 1A). Consequently, 200 μM of crocin was utilized for subsequent experiments. The atRAL-treated 661W cells shrunk and took on a spherical shape (Figure 1B). The treatment with crocin clearly ameliorated these morphological alterations (Figure 1B). Recent studies have highlighted the important role of oxidative stress in atRAL-induced damage in 661W cells [7,8,9,10]. Fluorescence microscopy with 2′,7′-dichlorodihydrofluorescein diacetate (H2DCFDA) staining showed that crocin effectively inhibited the generation of reactive oxygen species (ROS) in 661W cells treated with atRAL (Figure 1C,D). These findings indicate that crocin has the capacity to relieve damage and ROS generation caused by atRAL in photoreceptor cells.

### 2.2. Crocin Mitigates Mitochondrial Damage in atRAL-Loaded 661W Cells

**Figure 2:**
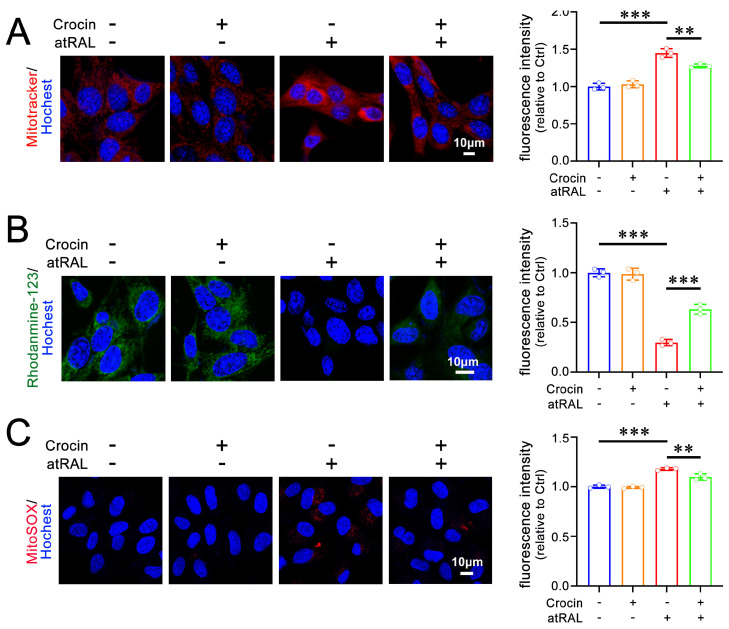
Protective effects of crocin on mitochondrial damage by atRAL in 661W cells. (**A**) Mitochondria were detected using Mitotracker^®^ Red CMXRos staining. (**B**) ΔΨm was examined by Rhodamine-123 staining. (**C**) Mitochondrial superoxide levels were determined by MitoSOX Red staining. Scale bars in (**A**–**C**) 10 μm. Fluorescence intensity in (**A**–**C**) was quantified by ImageJ software and is shown as fold changes relative to DMSO-treated controls. ** *p* < 0.01; *** *p* < 0.001.

Past studies have demonstrated that atRAL induces mitochondrial injury in 661W cells [7,8,9]. The distribution of mitochondria was analyzed using Mitotracker^®^ Red CMXRos staining. Compared to normal mitochondria that were evenly dispersed, mitochondria in atRAL-loaded 661W cells visibly aggregated within the cytoplasm (Figure 2A). Treatment with 200 μM of crocin clearly alleviated mitochondrial aggregation induced by atRAL in 661W cells (Figure 2A). The imaging of mitochondrial membrane potential (ΔΨm) with rhodamine-123 staining revealed that crocin significantly prevented an atRAL-induced decrease in ΔΨm levels in 661W cells (Figure 2B). In addition, mitochondrial superoxide imaging using MitoSOX Red staining showed that crocin remarkably attenuated an increase in mitochondrial superoxide levels in atRAL-treated 661W cells (Figure 2C). Taken together, these results suggest the ability of crocin to ameliorate mitochondrial damage by atRAL in photoreceptor cells.

### 2.3. Crocin Protects 661W Cells from Apoptosis Induced by atRAL

**Figure 3:**
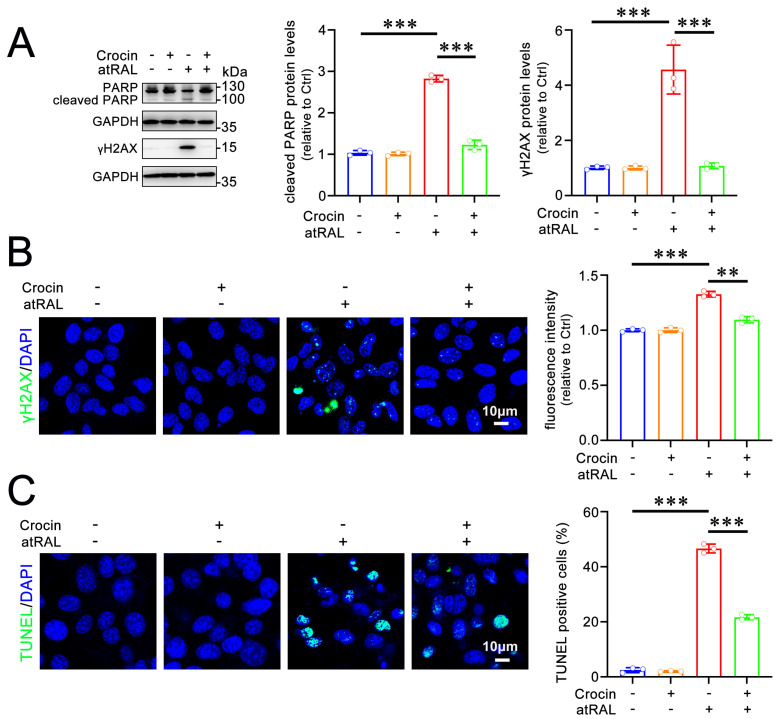
Crocin prevents apoptosis of atRAL-loaded 661W cells. (**A**) Western blots of PARP, cleaved PARP, and γH2AX. (**B**) Immunofluorescence analysis of γH2AX. (**C**) Apoptosis was measured by TUNEL staining. The percentage of TUNEL-positive cells was relative to the total number of cells. Scale bars in (**B**,**C**) 10 μm. ** *p* < 0.01; *** *p* < 0.001.

atRAL drives apoptosis in 661W cells via inducing DNA damage [7]. The protein levels of the phosphorylated histone H2A variant (γH2AX) and cleaved poly-ADP-ribose polymerase (PARP), markers of DNA damage during apoptosis, were significantly elevated in atRAL-loaded 661W cells (Figure 3A). Treatment with 200 μM of crocin distinctly reduced levels of cleaved PARP and γH2AX proteins in 661W cells after exposure to atRAL (Figure 3A). Immunofluorescence analysis revealed that a significant increase in levels of the γH2AX protein was observed in the nucleus of 661W cells treated with atRAL, and this was clearly prevented by crocin (Figure 3B). Therefore, crocin significantly repressed DNA damage in atRAL-treated 661W cells (Figure 3A,B). Additionally, the inhibitory effects of crocin on apoptosis were further evaluated by labeling apoptotic cells using the TUNEL assay. The TUNEL staining results demonstrated a clear elevation in the number of TUNEL-positive cells in atRAL-treated 661W cells, which was markedly blocked by crocin (Figure 3C). These data imply that crocin effectively mitigates apoptosis in atRAL-loaded photoreceptor cells.

### 2.4. Crocin Protects 661W Cells from Pyroptosis Induced by atRAL

**Figure 4:**
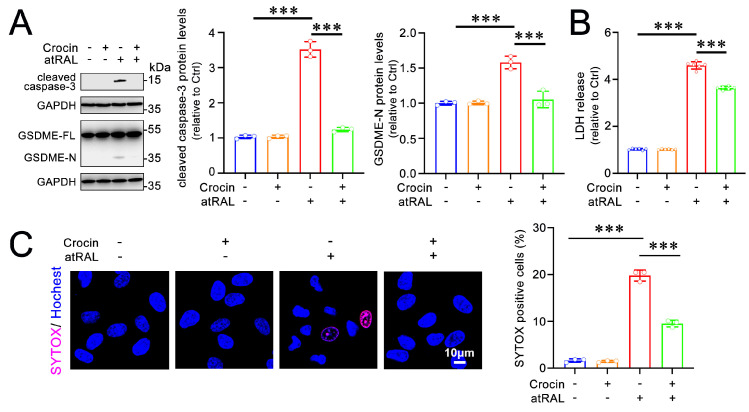
Crocin alleviates the pyroptosis of atRAL-loaded 661W cells. (**A**) Western blots of cleaved caspase-3, GSDME-FL, and GSDME-N. (**B**) LDH release detection. (**C**) The ruptured plasma membrane was detected by SYTOX staining. Scale bars, 10 μm. The percentage of SYTOX-positive cells is relative to the total number of cells. *** *p* < 0.001.

In addition to apoptosis, atRAL causes pyroptosis in 661W cells [9]. Gasdermins (GSDMs), a family of pore-forming effector proteins, are executors of pyroptosis [19,20]. A previous report demonstrated that the activation of GSDME, but not gasdermin D (GSDMD), induces pyroptosis in 661W cells exposed to atRAL [9]. The activation of GSDME by caspase-3 implies that the full-length form of GSDME (GSDME-FL) is cleaved to form the active N-terminal fragment of GSDME (GSDME-N) [9]. As expected, the protein levels of cleaved caspase-3 and GSDME-N were markedly elevated in atRAL-treated 661W cells. However, treatment with 200 μM of crocin significantly reduced the levels of cleaved caspase-3 and GSDME-N proteins in 661W cells following exposure to atRAL (Figure 4A). Pyroptosis usually leads to cell membrane rupture due to activated GSDMs forming pores in the membrane [21]. To further confirm that crocin alleviates pyroptosis in atRAL-treated 661W cells, the effects of crocin on plasma membrane integrity were evaluated using the lactate dehydrogenase (LDH) assay. The results showed that crocin significantly prevented the release of LDH from 661W cells following atRAL treatment (Figure 4B). In addition, the SYTOX staining of cells with disrupted plasma membranes revealed that treatment with crocin significantly decreased the number of SYTOX-positive cells (Figure 4C). These results indicate that crocin attenuates atRAL-induced pyroptosis in photoreceptor cells by inhibiting the formation of GSDME-N.

### 2.5. Crocin Protects 661W Cells from Ferroptosis Induced by atRAL

**Figure 5:**
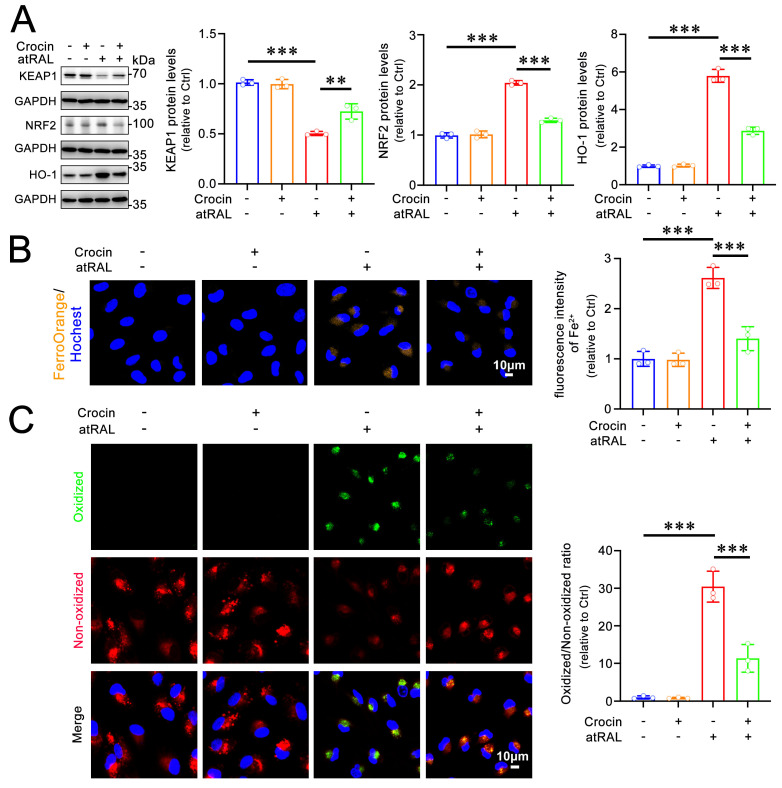
Crocin relieves the ferroptosis of atRAL-loaded 661W cells. (**A**) Western blots of KEAP1, NRF2, and HO-1. (**B**) Intracellular Fe^2+^ detection. (**C**) Lipid peroxidation measurement. The oxidized/non-oxidized fluorescence intensity ratios are presented as fold changes relative to DMSO-treated controls. Scale bars in (**B**,**C**) 10 μm. ** *p* < 0.01; *** *p* < 0.001.

Ferroptosis, a type of cell death that differs from apoptosis and pyroptosis, is primarily characterized by iron overload and lipid peroxidation [22,23]. Previous evidence has demonstrated that atRAL induces ferroptosis in 661W cells by activating the Kelch-like ECH-associated protein 1 (KEAP1)/nuclear factor-erythroid 2-related factor 2 (NRF2)/HO-1 signaling pathway [11]. A western blot analysis showed that 200 μM of crocin recovered KEAP1 degradation and inhibited the activation of NRF2 and HO-1 in 661W cells exposed to atRAL (Figure 5A). To further clarify whether crocin modulates ferroptosis in atRAL-treated 661W cells, the effects of crocin on iron overload and lipid peroxidation were examined. The confocal imaging following FerroOrange staining showed that treatment with crocin significantly reduced Fe^2+^ levels in 661W cells following exposure to atRAL (Figure 5B). The imaging of lipid peroxidation revealed that crocin significantly attenuated lipid peroxidation in atRAL-treated 661W cells (Figure 5C). These findings suggest that crocin is capable of inactivating the KEAP1/NRF2/HO-1 signaling pathway, thereby ameliorating atRAL-induced ferroptosis in photoreceptor cells.

## 3. Discussion

**Figure 6:**
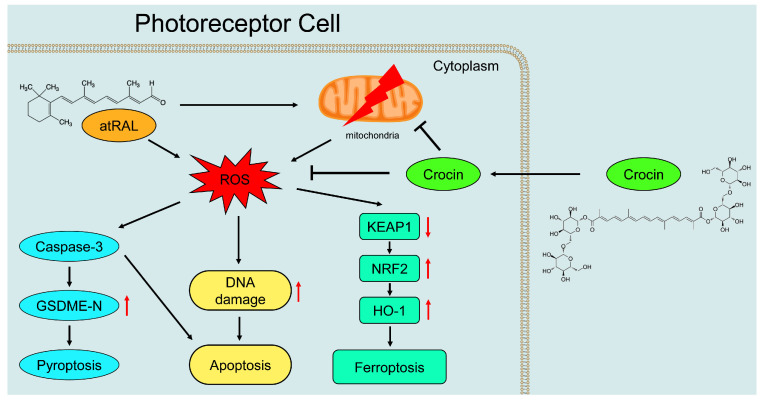
Schematic presentation of the mechanisms by which crocin protects photoreceptor cells against the toxicity of atRAL. Crocin attenuates the toxicity of atRAL in photoreceptor cells by inhibiting apoptosis, pyroptosis, and ferroptosis. atRAL, all-*trans*-retinal; ROS, reactive oxygen species; GSDME-N, N-terminal fragment of gasdermin E; KEAP1, Kelch-like ECH-associated protein 1; NRF2, nuclear factor-erythroid 2-related factor-2; HO-1, heme oxygenase-1. ↑, up-regulation; ↓, down-regulation.

The toxicity of atRAL in photoreceptors is one of the important features characterizing dry AMD pathogenesis, which can be utilized to evaluate the protective effects of crocin on atRAL-containing photoreceptor cells and explore its molecular mechanisms. Our findings revealed the following: (1) crocin ameliorated cell viability and morphology and reduced oxidative stress in atRAL-loaded photoreceptor cells; (2) crocin attenuated atRAL-induced mitochondrial damage by enhancing ΔΨm and decreasing the generation of mitochondrial superoxides in photoreceptor cells; (3) crocin mitigated photoreceptor cell apoptosis via alleviating DNA damage by atRAL; (4) crocin inhibited the photoreceptor cell pyroptosis caused by atRAL through inactivating the caspase-3/GSDME signaling pathway; (5) crocin alleviated the ferroptosis of photoreceptor cells exposed to atRAL by regulating the KEAP1/NRF2/HO-1 signaling pathway. Based on the foregoing results, we illustrate the molecular mechanisms by which crocin protects photoreceptor cells, as summarized in Figure 6.

Chemical inducers, such as hydrogen peroxide and sodium iodate, are often used to establish experimental cell models to study photoreceptor cell damage [24,25]. However, compared to photoreceptor cell damage models created by chemical inducers, a photoreceptor cell damage model established by atRAL toxicity better represents AMD pathogenesis [26,27]. AMD patients commonly suffer from visual cycle disorders, which cause the accumulation of atRAL in photoreceptors, ultimately leading to retinal degeneration [28,29]. Several lines of investigation have shown that toxic atRAL evokes mitochondrial injury and ROS production, which promotes photoreceptor cell damage [30,31].

Crocin is a natural product extracted from saffron that has multiple biological functions [32,33]. It modulates oxidative stress within the nervous system and has protective effects against various organ injuries [34,35,36]. Saffron extracts protect retinal ganglion cells by decreasing retinal microglial activation, which may serve as a functional ingredient for glaucoma [16]. Crocin also rescues primary bovine photoreceptors from damage by light through inhibiting apoptosis [37]. Furthermore, a clinical trial found that crocin serves as a supplement to reduce retinal inflammation in patients with diabetic maculopathy [18]. Although these studies have confirmed the protective effects of crocin on the retina, the effects of crocin on atRAL-loaded photoreceptor cells and its mechanisms remain unclear. In this study, we demonstrated that crocin mitigated atRAL-induced damage in photoreceptor cells by inhibiting oxidative stress, apoptosis, pyroptosis, and ferroptosis.

Apoptosis is a programmed cell death process marked by chromatin condensation and DNA fragmentation [38,39]. Apoptotic cells exhibit both DNA damage and oxidative stress, ultimately contributing to their death [40,41]. Our findings reveal that crocin significantly inhibits atRAL-induced apoptosis in photoreceptor cells by reducing DNA damage and oxidative stress. Previous reports have demonstrated that the activation of eukaryotic translation initiation factor 2α (eIF2α) or c-Jun N-terminal kinase (JNK) elicits the photoreceptor cell apoptosis induced by atRAL [7,8]. As expected, treatment with 200 µM of crocin suppressed the activation of eIF2α and JNK by atRAL (Figure S1). Pyroptosis is a form of cell death associated with the gasdermins (GSDMs) family of pore-forming proteins [42,43]. The cleaved N-terminal fragments of GSDMs create pores in the cell membrane, leading to cell swelling and membrane rupture [44,45]. In this investigation, crocin effectively reduced the cleavage of GSDME by repressing the activity of caspase-3, thereby resisting the photoreceptor cell pyroptosis caused by atRAL. Ferroptosis is a regulated form of cell death characterized by iron overload and lipid peroxidation [46,47]. HO-1 plays a crucial role in ferroptosis, which is largely regulated by the KEAP1/NRF2 signaling pathway [48,49]. In the current study, crocin upregulated KEAP1 activity to inhibit NRF2 expression, thereby inhibiting HO-1-induced ferroptosis in photoreceptor cells exposed to atRAL. Collectively, these findings provide evidence that crocin relieves atRAL-induced apoptosis, pyroptosis, and ferroptosis to facilitate photoreceptor cell survival. However, it is necessary to further verify the protective effects of crocin on atRAL-induced damage in photoreceptors through animal experiments and clinical trials.

In conclusion, crocin effectively reduces oxidative stress and mitigates apoptosis, pyroptosis, and ferroptosis in photoreceptor cells exposed to atRAL, which suggests that it may serve as a promising antioxidant ingredient to alleviate atRAL toxicity and prevent photoreceptor cell damage.

## 4. Materials and Methods

### 4.1. Reagents and Antibodies

atRAL (catalog no. R2500), Hoechst 33342 (catalog no. B2261), and 4′,6-diamidino-2-phenylindole (DAPI) (catalog no. F6057) were purchased from Sigma–Aldrich (Saint-Louise, MI, USA). Crocin (catalog no. HY-N0697) was obtained from MedChemExpress (Shanghai, China). FerroOrange (catalog no. F374) was purchased from DOJINDO (Kyushu, Japan). 2′,7′-dichlorodihydrofluorescein diacetate (H2DCFDA) (catalog no. D399), rhodanmine-123 (catalog no. R302), MitoSOXTM Red mitochondrial superoxide indicator (catalog no. 36008), SYTOX Stain Kit (catalog no. S34826), and image-iT^TM^ lipid peroxidation kit (catalog no. C10445) were purchased from Thermo Fisher Scientific (Rockford, IL, USA). Antibodies against cleaved caspase-3 (catalog no. 9664S), PARP (catalog no. 9542S), KEAP1 (catalog no. D6B12), NRF2 (catalog no. D1Z9C), HO-1 (catalog no. E6Z5G), eIF2α (catalog no. 9722S), p-eIF2α (catalog no. 3398S), p-JNK (catalog no. 9255S), and GAPDH (catalog no. 5174S) were provided by Cell Signaling Technology (Danvers, MA, USA). Anti-γH2AX (catalog no. 05-636) was purchased from Sigma–Aldrich (Saint-Louise, Missouri, USA). Anti-GSDME (catalog no. ab215191) was purchased from Abcam (South Cambs, England, UK). Alexa Fluor 488-conjugated donkey anti-mouse IgG (H + L) (catalog no. A21202), anti-rabbit IgG (H + L) (catalog no. 31460), and anti-mouse IgG (H + L) (catalog no. A21203) secondary antibodies were purchased from Thermo Fisher Scientific (Rockford, IL, USA).

### 4.2. Cell Culture

The 661W murine photoreceptor cell line was sourced from Shanghai Zishi Biotechnology (Shanghai, China). Cells were cultured in Dulbecco’s modified Eagle’s medium (DMEM) (Gibco, Beijing, China) supplemented with 1%(*v*/*v*) penicillin-streptomycin (Biosharp, Bengbu City, China) and 10% (*v*/*v*) fetal bovine serum (Wisent, Saint-Jean-Baptiste, QC, Canada). Cells were cultured in an incubator with 5% CO_2_ and saturated humidity at 37 °C.

### 4.3. Cell Viability

Cell viability was evaluated using the MTS assay (Promega, WI, USA) [7]. 661W cells were preincubated with serial concentrations of crocin (50, 100, or 200 μM) for 2 h, followed by 6 h of treatment with 5 μM atRAL. Cytotoxicity was assessed by adding 120 μL of the detection reagent directly to the wells, followed by a 1 h incubation period. Absorbance was measured at 490 nm using a Multiskan GO-1510 spectrophotometer (Thermo Fisher Scientific).

### 4.4. Detection of ROS

661W cells seeded in 6-well plates were preincubated with 200 μM crocin for 2 h, followed by 6 h of treatment with 5 μM atRAL. Subsequently, cells were incubated with 10 μM H2DCFDA and 10 μM Hoechst 33342 for 30 min at 37 °C. After washing three times with phosphate-buffered saline (PBS), 1 mL of fetal bovine serum (FBS)-free DMEM was added to each well. Cells were then observed under a DMi8 Leica fluorescence microscope (Leica Microsystems; Wetzlar, Germany). Each confocal image was acquired by capturing fluorescence from a specific focal plane where ROS signals were most pronounced.

### 4.5. Staining of Mitochondria

Upon exposure of atRAL to 661W cells with or without crocin treatment, cells were incubated with Mitotracker^®^ Red CMXRos (500 nM), rhodamine-123 (10 mg/mL), or MitoSOX (5 μM) for 30 min at 37 °C. After washing three times with PBS, 1 mL of fresh DMEM was added to each well. Cells were photographed using a Zeiss LSM 880 confocal microscope (Carl Zeiss; Jena, Germany).

### 4.6. TUNEL Assay

Apoptosis was assessed using a TUNEL assay (Promega; WI, USA) [7]. Slides were stained with DAPI. Images were captured using the Zeiss LSM 880 confocal microscope.

### 4.7. LDH Assay

LDH release was determined using an LDH cytotoxicity assay kit (Yeasen; Shanghai, China) [9]. In total, 60 μL of the detection reagent for LDH release was added to each well for 1 h. The plates were then centrifuged at 400× *g* for 5 min, and 120 μL of the supernatant from each well was taken for analysis using the Multiskan GO-1510 spectrophotometer.

### 4.8. SYTOX Staining

SYTOX Dead Cell assay was used to detect compromised plasma membranes of cells [21]. Cells were incubated with 10 μM SYTOX and 10 μM Hoechst 33342 at 37 °C for 30 min and then washed three times with PBS. Imaging was performed using the Zeiss LSM 880 confocal microscope.

### 4.9. Detection of Fe^2+^ Levels

Cells were treated with 1 μM FerroOrange and 10 μM Hoechst 33342 for 30 min at 37 °C and then washed three times with PBS. Imaging was performed using the Zeiss LSM 880 confocal microscope.

### 4.10. Detection of Lipid Peroxidation

The Image-iT™ lipid peroxidation kit was utilized to assess cellular lipid peroxidation [11]. Cells were incubated for 40 min with 10 μM C11-BODIPY 581/591 and 10 μM Hoechst 33342 at 37 °C and then washed with PBS three times. Imaging was performed using the Zeiss LSM 880 confocal microscope.

### 4.11. Immunofluorescence Staining

Cells were fixed with 4% paraformaldehyde for 15 min and permeabilized by 0.2% Triton X-100 in PBS for 20 min at room temperature. Next, cells were blocked with 2% bovine serum albumin (BSA) and incubated with γH2AX primary antibodies (1:100 dilution) overnight at 4 °C, followed by incubation with 488-conjugated secondary antibodies (1:200 dilution) for 1 h at room temperature. Slides were mounted in Vectashield with DAPI (Vector Labs; Burlingame, CA, USA). Imaging was performed using the Zeiss LSM 880 confocal microscope.

### 4.12. Western Blotting

Western blot analysis of cell extracts was carried out as previously described [11]. Membranes were incubated overnight at 4 °C with a primary rabbit antibody (1:1000) specific for PARP, γH2AX, cleaved caspase-3, GSDME, KEAP1, NRF2, HO-1, eIF2α, p-eIF2α, p-JNK, or GAPDH. The membranes were then incubated with the goat-anti-rabbit secondary antibody for 2 h at room temperature. Protein bands were visualized using a ChemiDoc XRS+ Imaging system (Bio-Rad; Hercules, CA, USA) with ECL Western blotting detection reagents (Advansta; Menlo Park, CA, USA). The levels of each protein were normalized to those of GAPDH and presented as fold changes relative to DMSO-treated controls. Uncropped original images of gels, in which boxes in red indicated selected Western blot results, are shown in Figure S2.

### 4.13. Statistical Analyses

All data were analyzed using GraphPad Prism software (Version 8.0). Results are presented as the mean ± standard deviation (SD) of at least three independent experiments. Statistical analyses were conducted using one-way ANOVA followed by Tukey’s multiple comparison test. In all cases, *p*-values below 0.01 were considered statistically significant. ** *p* < 0.01; *** *p* < 0.001.

## Data Availability

The data presented in this study are available on request from the corresponding author.

## Supplementary Materials

The following supporting information can be downloaded at https://www.mdpi.com/article/10.3390/ijms251810124/s1.
