# Supplementary material for: Crocin Protects the 661W Murine Photoreceptor Cell Line against the Toxic Effects of All-Trans-Retinal
Source: Int J Mol Sci. 2024 Sep 20;25(18):10124. doi: 10.3390/ijms251810124 (PMC11432120; doi:10.3390/ijms251810124)

Supplementary Information

Crocin protects the 661W murine photoreceptor cell line against the toxic effects of all-*trans*-retinal

Bo Yang [1], Kunhuan Yang [1], Jingmeng Chen [2,3], Yalin Wu [1,3,*]

[1] Fujian Provincial Key Laboratory of Ophthalmology and Visual Science, Fujian Engineering and Research Center of Eye Regenerative Medicine, Eye Institute of Xiamen University, School of Medicine, Xiamen University, Xiamen, Fujian 361102, China

[2] School of Medicine, Xiamen University, Xiamen, Fujian 361102, China

[3] Shenzhen Research Institute of Xiamen University, Shenzhen, Guangdong 518057, China

*Correspondence: yalinw@xmu.edu.cn

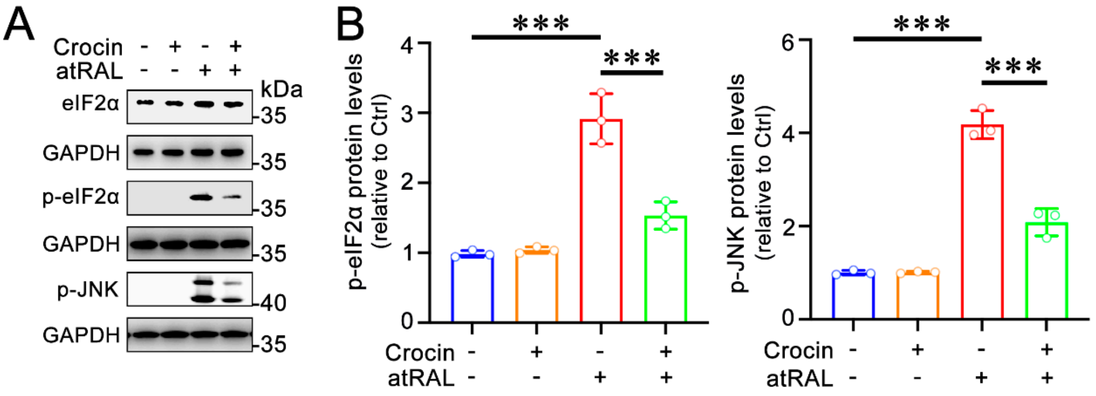

Supplementary Figure S1. Effect of crocin on the activation of eIF2α and JNK in atRAL-loaded 661W cells.

661W cells were pretreated with 200 μM crocin for 2 h and then incubated with 5 μM atRAL for 6 h. (A) Western blots of eIF2*α*, phosphorylated eIF2*α* (p-eIF2*α*) and phosphorylated JNK (p-JNK). (B) Protein levels of p-eIF2*α* and p-JNK were normalized to those of GAPDH, and presented as fold changes relative to vehicle (DMSO)-treated controls. Each value represents the mean ± SD of three independent experiments. Statistical analyses were performed by using one-way ANOVA with Tukey's multiple comparison test. ***p<0.001.

Supplementary Figure S2. Uncropped western blots.

Boxes in *red* indicate selected Western blot results.

Fig. 3A

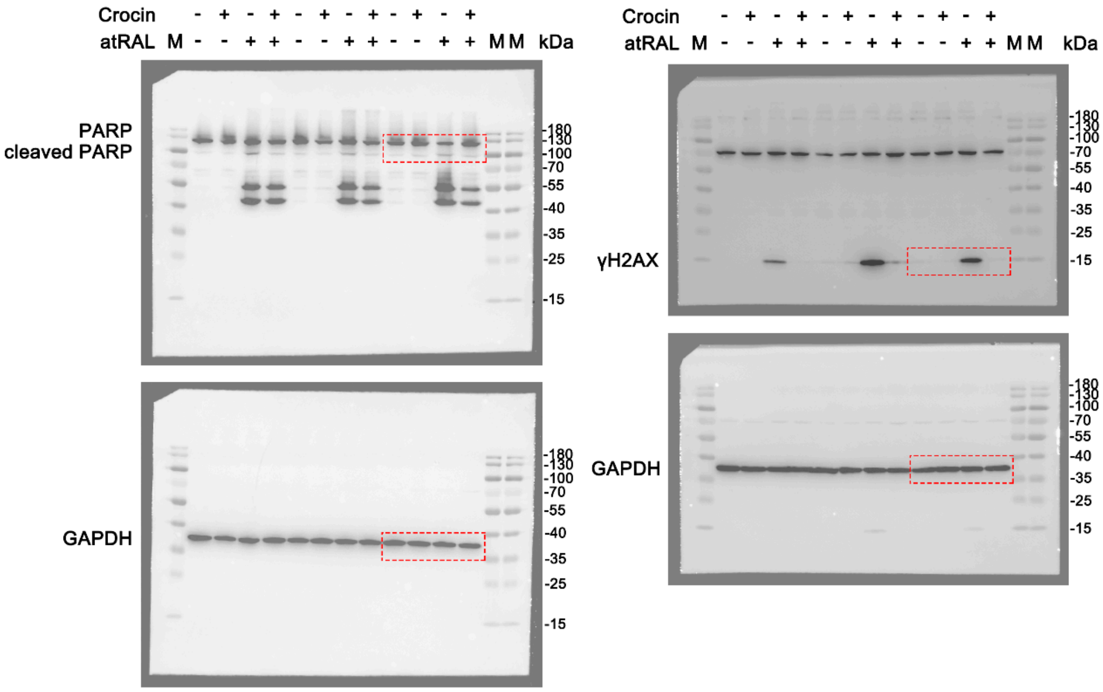

Fig. 4A

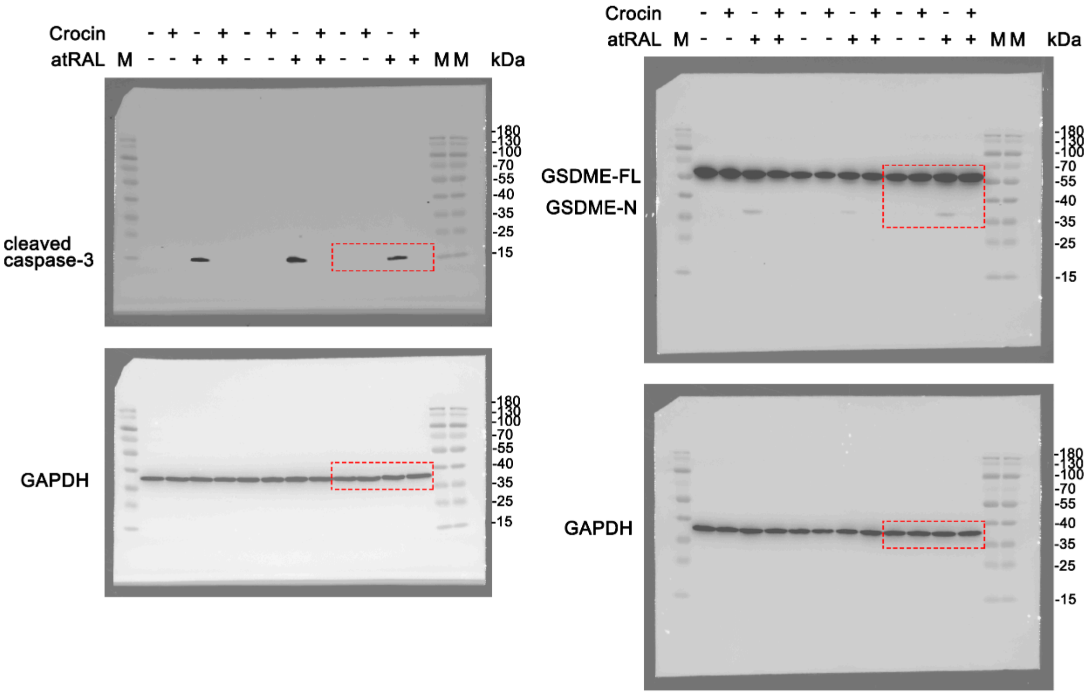

Fig. 5A

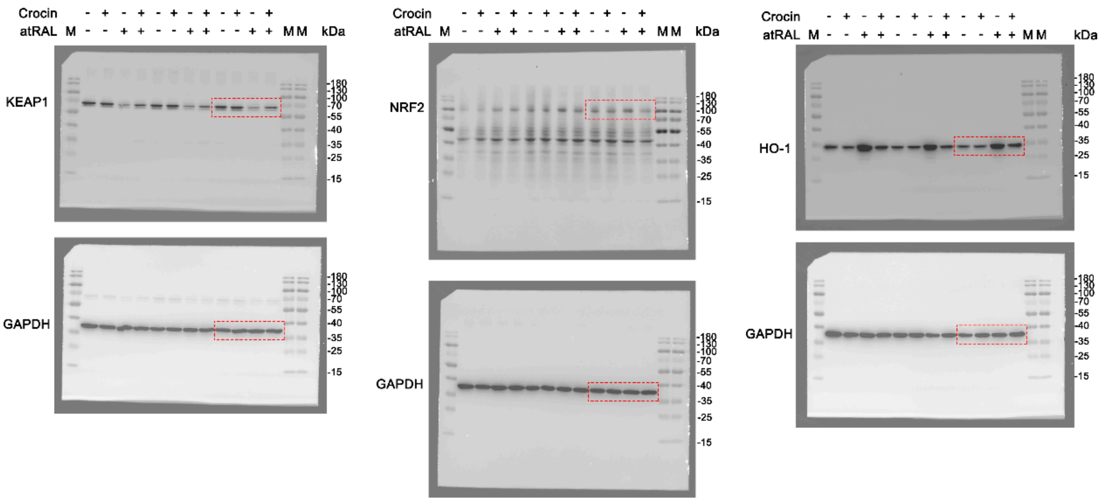

Fig. S1*A*

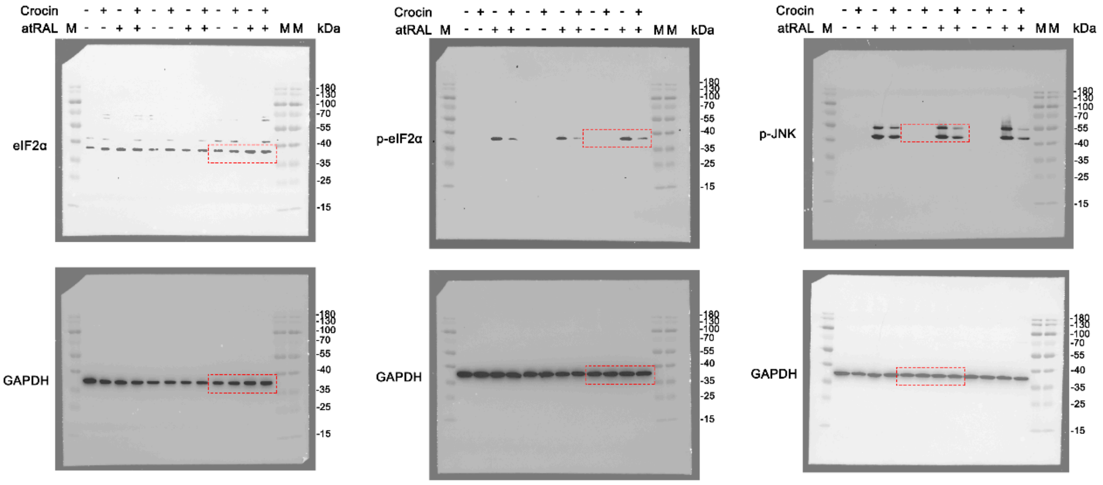

Supplement: Supplementary file 1 [file ijms-25-10124-s001.zip › ijms-3158363-supplementary.pdf]
